# Super-silent FRET Sensor Enables Live Cell Imaging and Flow Cytometric Stratification of Intracellular Serine Protease Activity in Neutrophils

**DOI:** 10.1038/s41598-018-31391-9

**Published:** 2018-09-10

**Authors:** Thomas H. Craven, Nicolaos Avlonitis, Neil McDonald, Tashfeen Walton, Emma Scholefield, Ahsan R. Akram, Timothy S. Walsh, Chris Haslett, Mark Bradley, Kevin Dhaliwal

**Affiliations:** 1 EPSRC Proteus IRC, MRC Centre for Inflammation Research, University of Edinburgh, EH16 4TJ Edinburgh, United Kingdom; 2 EaStChem, School of Chemistry, University of Edinburgh, King’s Buildings, EH9 3JN Edinburgh, United Kingdom; 3 MRC Centre for Inflammation Research, University of Edinburgh, EH16 4TJ Edinburgh, United Kingdom; 4 Anaesthetics, Critical Care and Pain Medicine, Royal Infirmary of Edinburgh, EH16 4SA Edinburgh, United Kingdom

## Abstract

Serine proteases are released by neutrophils to act primarily as antimicrobial proteins but excessive and unbalanced serine protease activity results in serious host tissue damage. Here the synthesis of a novel chemical sensor based on a multi-branched fluorescence quencher is reported. It is super-silent, exhibiting no fluorescence until de-quenched by the exemplar serine protease human neutrophil elastase, rapidly enters human neutrophils, and is inhibited by serine protease inhibitors. This sensor allows live imaging of intracellular serine protease activity within human neutrophils and demonstrates that the unique combination of a multivalent scaffold combined with a FRET peptide represents a novel and efficient strategy to generate super-silent sensors that permit the visualisation of intracellular proteases and may enable point of care whole blood profiling of neutrophils.

## Introduction

Human neutrophil elastase (HNE), Proteinase 3 and Cathepsin G are serprocidins (serine proteases with microbicidal activity) and are stored in active form in the primary azurophilic granules of human neutrophils^1^. Serprocidins provide host defence against bacterial infections as well as inducing activation of endothelial and epithelial cells, macrophages, lymphocytes, and platelets^2^. HNE is the most abundant serine protease, stored at millimolar concentrations in the primary granules of the neutrophil^3^. Injurious stimuli cause the neutrophil to undergo a range of physical actions including phagocytosis and degranulation. Phagocytosis leads to ingestion and destruction of invading microorganisms and degranulation causes digestion of invading microorganisms and their products and modulates the host inflammatory response. However, serprocidins display proteolytic activity against a variety of extracellular matrix components, such as elastin, fibronectin, laminin, type IV collagen, and vitronectin^1^ and excessive release from neutrophils has been implicated in pathophysiological conditions such as acute respiratory distress syndrome (ARDS), bronchiectasis, emphysema, and sepsis^4–8^. Whilst routine methodologies exist for the detection and quantification of individual seprocidins both in the laboratory and in the clinic, many of these rely on the antigenic detection of extracellular enzymes. There are two main weaknesses with this assay strategy. Firstly, *in vivo*, a range of endogenous inhibitors such as elafin and secretory leukocyte protease inhibitor (SLPI)^9^ are widely present and so antigenic quantification of seprocidins can be misleading. Secondly, these assays provide no spatiotemporal information regarding the activity of serprocidins either intra or extracellularly. For these reasons a serprocidin sensor for interrogating the spatial and temporal arrangement of enzyme activity in both the intra and extracellular environment could provide advantages over established detection methods. Such sensors could enable the assessment of neutrophil phenotype and novel anti-inflammatory pharmaceutical agents as well as providing opportunities for the stratification of patients suffering from a range of acute and chronic inflammatory conditions.

**Figure 1:**
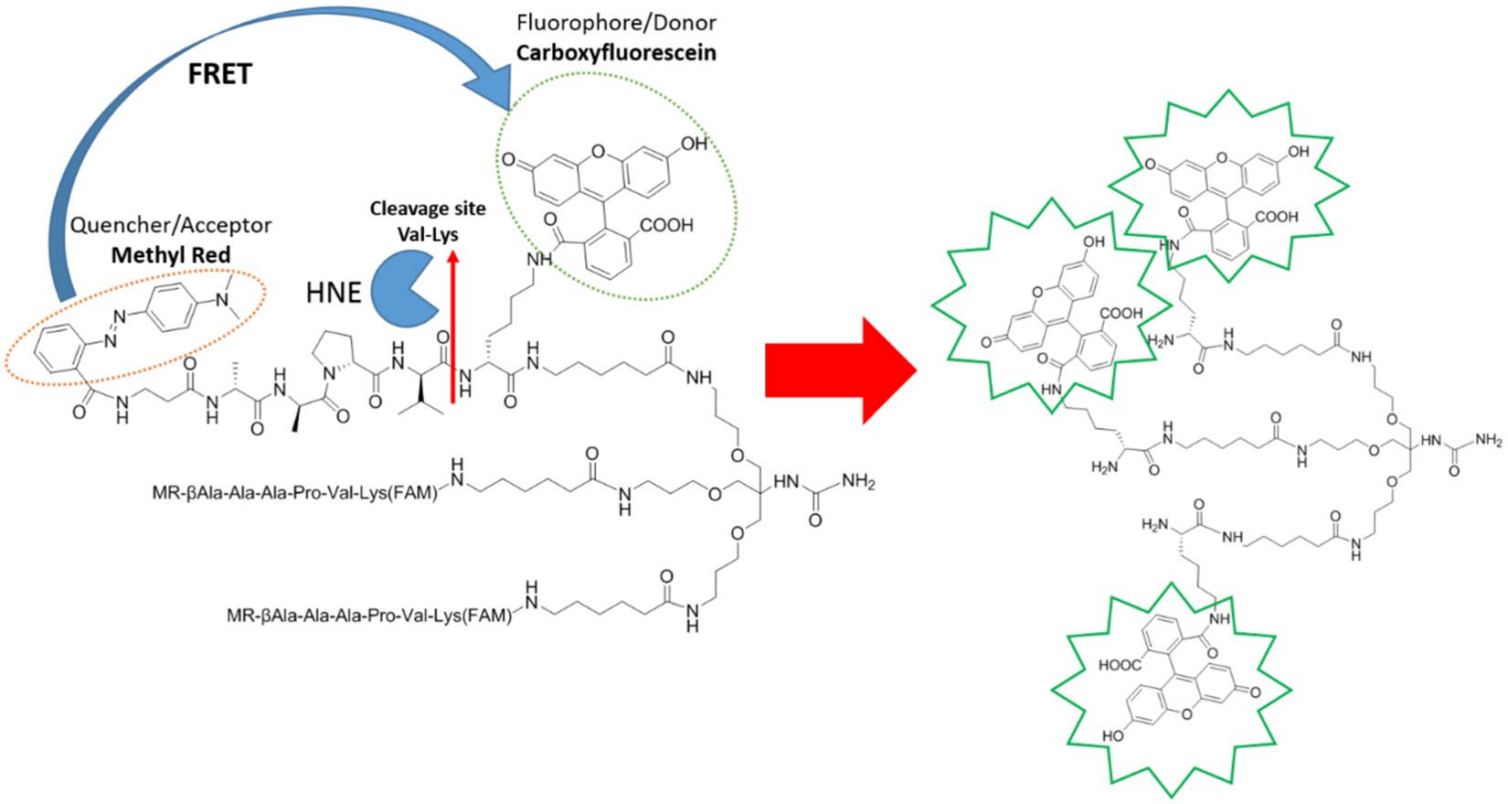
The tribranched scaffold utilizes a multi-branched FRET system that remains super-silent in the absence of enzyme. The trimeric structure facilitates vesicular uptake into neutrophils and under the activity of serine proteases the peptide sequence is hydrolysed releasing the methyl red quencher, where upon the fluorescein moieties fluoresce. Q = FRET Quencher, FAM = 5-carboxyfluorescein amide.

Classically, seprocidin activity has been investigated using peptidic substrates in which the *C*-terminus is conjugated to a chromogenic or fluorogenic group, which is released upon cleavage. The most commonly used fluorogenic substrate is *N-*(Methoxysuccinyl)-AAPV-4-methyl-7-coumaryl amide^10^. Alterations of the peptidic substrate have shown improved specificity for individual seprocidins; Gauthier has described HNE optimised FRET peptides such as Abz-APEEIMRRQ-EDDnp (Abz: 2-aminobenzoyl, EDDnp: *N*-(2,4-dinitrophenyl)ethylenediamine) as substrates for the measurement of HNE proteases on the surfaces of neutrophils^11^. FRET-based probes based on the AAPV substrate, as well as fluorescently-labelled inhibitors, have been used to visualise regional elastase activity *in vivo*^12,13^. Multivalent fluorescent peptides have demonstrated high biocompatibilities, low toxicities and an ability to access intracellular compartments^14^, and we previously reported on a lead optimised peptidic substrate incorporated into a multi-branched fluorescently labelled reporter to monitor HNE derived from human neutrophils^15^. This offered a single auto-quenching fluorophore based approach for the analysis of proteolytic activity, where amplification of signal upon substrate cleavage was possible, without needing to rely on two dyes, but demonstrated high background levels. Based on these observations, we have now designed a multivalent FRET peptide scaffold to give a *super-silent quenched sensor* with triple horizontal quenching, enabling the visualisation of intracellular and extracellular protease activity (Fig. 1). To demonstrate proof of concept of this novel scaffold, we have used the generic AAPV peptidic substrate with potential to iterate this peptide sequence to provide additional enzyme specificity.

## Results

### Probe synthesis

**Figure 2:**
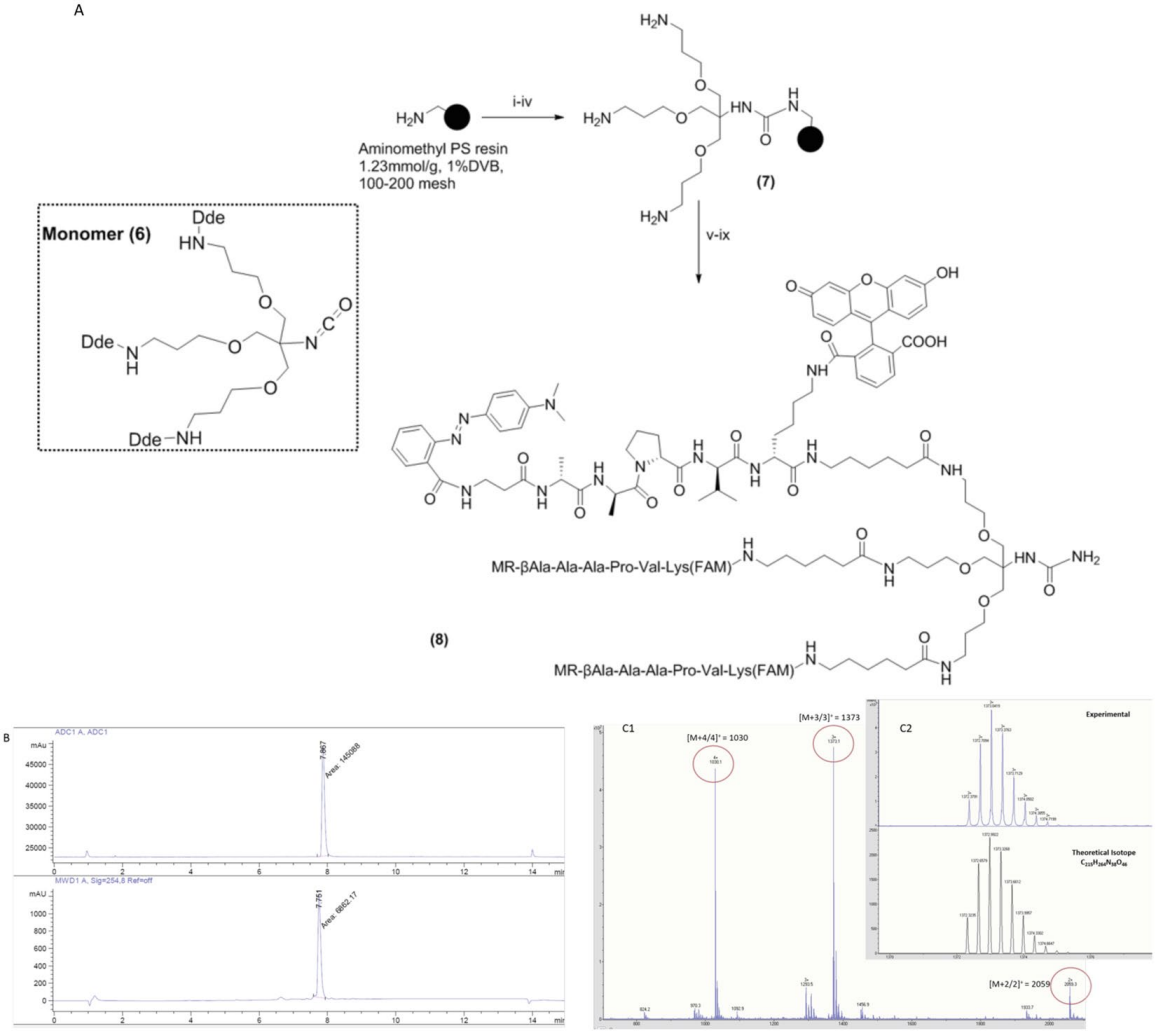
**(A)** Probe synthesis. Reagents and conditions: (i) Fmoc-Rink amide linker, HOBt, DIC, DMF; (ii) 20% Piperidine/DMF; (iii) Monomer (**6**), DIPEA, DMAP, DCM/DMF; (iv) 2% Hydrazine/DMF; (v) [(a) Fmoc-AA-OH, Oxyma, DIC, DMF, (b) 20% piperidine/DMF x 6]; (vi) Methyl red, Oxyma, DIC, DMF,; (vii) (a) 2% Hydrazine/DMF, b) 5(6)-carboxyfluorescein, Oxyma, DIC, DMF; (ix) TFA/DCM/TIS (95/2.5/2.5). MR = Methyl red, FAM = 5(6)-carboxyfluorescein amide, Ahx = 6-aminohexanoic acid, Dde: N-(1-(4,4-dimethyl-2,6-dioxocyclohexylidene)ethyl). **(B)** RP-HPLC chromatogram of probe (**8**) on a Discovery C18 reverse-phase column (50 × 4.6 mm, 5 μm) with a flow rate of 1 mL/min and eluting with 0.1% HCOOH in H_2_O **(A)** and 0.1% HCOOH in CH_3_CN **(B)**, a gradient of 5 to 95% B over 13 min and an initial isocratic period of 2 min with detection at 254 nm (lower) and by evaporative light scattering (upper); **(C)** FTMS analysis: *m/z* 1030 [M + 4/4]^+^, 1373 [M + 3/3]^+^ and 2059 [M + 2/2]^+^; insert spectral zoom (experimental and theoretical [M + 3/3]^+^).

The first step in the synthesis of the probe was the preparation of monomer (6) which was synthesised in six steps in an overall yield of 15% (supplementary materials)^15^. This was attached to a Knorr/Rink type-linker on an aminomethyl polystyrene resin, and followed by standard Fmoc-based solid phase peptide synthesis to build the peptide (Fmoc-Ala-Ala-Pro-Val-Lys(Dde) on the resin. β-Alanine was used as an amino-terminal spacing element and functionalised with methyl red, while the Lysine side chain was functionalised with 5(6)-carboxyfluorescein, followed by standard acidolysis to enable linker cleavage. The synthesis and analysis of the probe are shown in Fig. 2.

### Sensing of extracellular elastase

**Figure 3:**
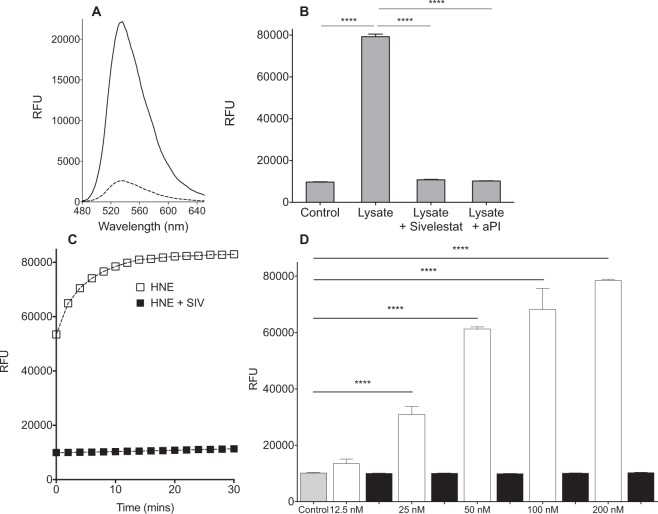
**(A)** Comparison of the fluorescence spectra of probe **(8**) (5 µM) (dashed line) after treatment with PMN lysate (solid line). **(B)** Incubation of PMN lysate (2.5 × 10^5^ cells/ml) leads to an increase in probe fluorescence that is inhibited by the presence of Sivelestat (100 µM) or by the endogenous HNE inhibitor αPI (200 µg/ml). **(C)** The time-course of probe de-quenching following incubation with purified HNE. Incubation with HNE leads to a rapid increase in fluorescence that is inhibited by the HNE specific inhibitor, Sivelestat (100 µM). X-axis refers to time on plate reader, several seconds after addition of Probe **(8)**, by which time significant de-quenching has occurred. **(D)** Analysis of the fluorescence increase after 10 minutes with (black columns) or without (white columns) Sivelestat (100 µM) at a range of concentrations of HNE. Statistical analysis by one-way ANOVA with Bonferroni’s post-hoc correction, ****P < 0.0001.

Probe **(8)** exhibited very low background fluorescence (Fig. 3A). As the main serine protease available *in* vivo, we use HNE as the primary enzyme for examination *in vitro*. When treated with purified HNE, the probe demonstrated a significant increase in fluorescence within seconds with a 10-minute Limit of Detection (LoD) of 10 nM (Fig. 3C) and showed fluorescence increases in a dose dependent fashion as the concentration of purified HNE increased over the physiologically relevant range^16^. Incubation with PMN lysate led to a rapid increase of fluorescence (8.5 fold – Fig. 3B). Primary human PMN lysates are abundant in metalloproteases, reactive oxygen species and serine proteases. To demonstrate the selective activation of the probe by neutrophil derived serine proteases, lysates were pre-incubated with alphaPI (known to inhibit HNE, PR3, and Cathepsin G), and Sivelestat (Fig. 3B); and showed total inhibition of probe cleavage. This demonstrates that probe **(8)** is selective for serine proteases and is not cleaved by non-serine protease enzymes.

### Intracellular serprocidin sensing in neutrophils

**Figure 4:**
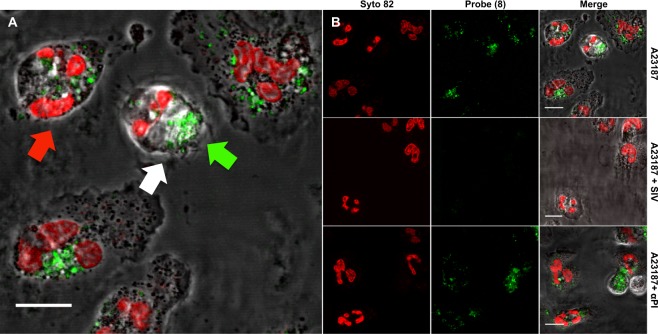
**(A)** Addition of probe **(8)** (5 µM) to freshly isolated human PMNs that have been pharmacologically activated by the calcium ionophore A23187 (10 µM) leads to the rapid appearance of a punctate cell-associated green fluorescence signal (via cleavage of AAPV) when excited at 488 nm. The signal of the probe (green arrow) is observed in the intracellular confocal plane of the PMN nucleus (red arrow) labelled by the fluorescent DNA dye Syto-82 (red) and within the cell boundaries (white arrow). Scale bar: 10 µm. Images are representative of 3 independent experiments. **(B)** Activation of freshly isolated human PMN with A23187 (10 µM) leads to the rapid appearance of a punctate cell-associated fluorescence signal from the probe **(8)** when imaged by laser-scanning confocal microscopy (shown at t + 10 minutes). Syto 82 labels the PMN nucleus. Pre-treatment (10 mins) with sivelestat (100 µM) inhibits the appearance of this punctate cell-associated fluorescence whereas pre-treatment with αPI (200 µg/ml) does not. PMN were imaged live in the continued presence of probe **(8)** and inhibitors. Scale bar: 10 µm. Images are representative of 3 independent experiments.

**Figure 5:**
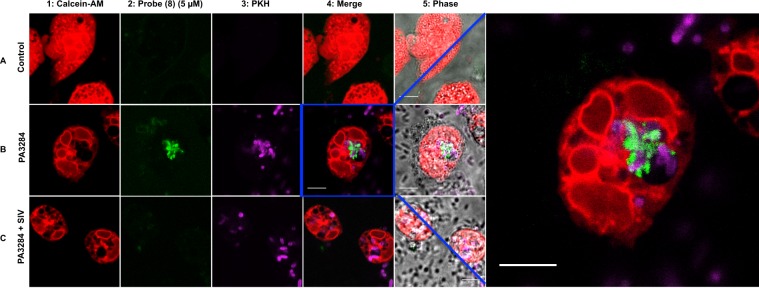
Live cell laser-scanning confocal microscopy of human PMN following the addition of the probe (**8**) (5 µM). Non-activated neutrophils are seen in row **A**. Rows **B** and **C** depict neutrophils that have been activated by co-culture with *Pseudomonas aeruginosa* (J3284). Row **C** neutrophils were additionally co-cultured in the presence of Sivelestat (100 µM). Column 1: Extensive vesicular formation is seen in neutrophils stained with Calcein-AM. Probe (**8**) fluorescence (green) signal (column 2) is seen in activated neutrophils but is absent in quiescent neutrophils or in the presence of Sivelestat. The merged images (column 4) show the co-localisation of labelled bacteria (PKH) and probe (**8**) signal within a neutrophil vesicle. Far right panel shows magnified image of co-localised bacteria and probe (**8**) signal. Scale bar: 5 µm. Images are representative of fields of view from three independent experiments.

Intracellular seprocidin activity is required for killing of bacteria within the neutrophil^3,17–19^. Based on the propensity of dendrimeric scaffolds to carry cargoes into cells^20,21^, the intracellular visualisation of serprocidin activity was explored. To investigate intracellular serprocidin activity, freshly isolated primary human PMNs were either activated pharmacologically using the calcium ionophore A23187 (Fig. 4), or co-cultured with a clinical strain of *Pseudomonas aeruginosa* (Fig. 5). Cells were imaged with confocal microscopy, demonstrating a punctate fluorescent signal appearing throughout the cytoplasm within seconds of activation. This demonstrated that the multivalent scaffold efficiently and rapidly entered cells where it was able to rapidly report on intracellular serprocidin activity in live cells. The punctate fluorescent cellular signal was abrogated by the addition of Sivelestat, which is known to be cell-permeable^22^. The anti-protease αPI is not cell permeable and did not inhibit this signal, thereby showing that the origin of intra-cellular fluorescence is not endocytosis of cleaved probe, but of probe that has been internalised and cleaved (and de-quenched) (Fig. 4).

To characterise further the intracellular localization of fluorescent signal, freshly isolated neutrophils were stained with the cytoplasmic dye (Calcein-AM) to reveal sites of vesicle formation, which were readily visualized by the lack of cytoplasmic dye, and exposed to labelled bacteria (*Pseudomonas aeruginosa* (Fig. 5C)). Live confocal microscopy revealed the co-localization of labelled bacteria and probe (**8**) within the phagolysosome confirming the intravacuolar activity of the serprocidins^23^.

### Probe (8) rapidly profiles activated neutrophils in whole blood using flow cytometric analysis

**Figure 6:**
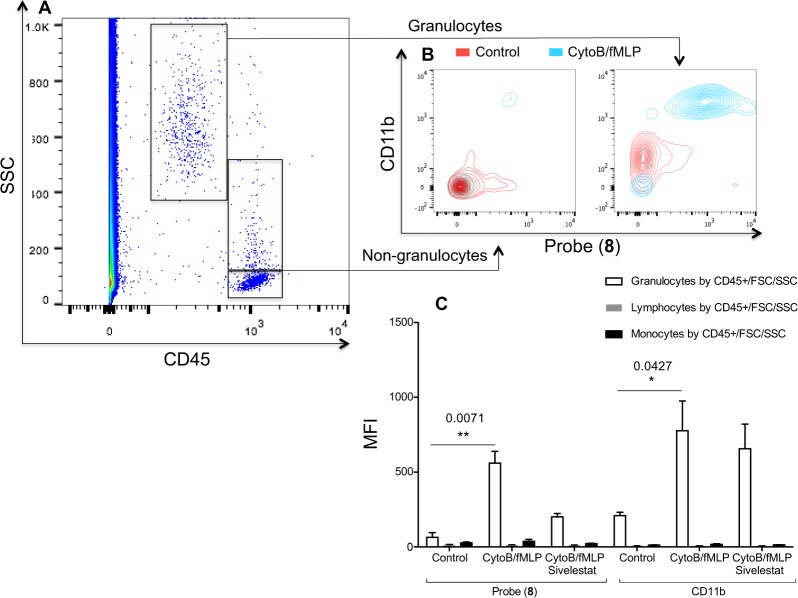
Aliquots of whole human blood were activated with Cytochalasin B and fMLP with or without Sivelestat or left untreated. 10 µL of whole blood was diluted 1:10 into IMDM, which contained the appropriate concentrations of antibodies. Following labelling as described, Probe (**8**) was applied at 5 µM for 10 minutes at 22 °C before the samples were again diluted into 1.4 mL of IMDM. **(A)** CD45 positive cells were separated into discrete populations of leucocytes according to their light scatter characteristics. Only events that were appropriately located on a FSC/SSC were forwarded for analysis (plot not shown). **(B)** Non-granulocytes did not take up Probe (**8**) or demonstrate an up regulation of CD11b. Quiescent granulocytes exhibited the same characteristics but activated granulocytes were +/+ for Probe (**8**)/CD11b. **(C)** Activated granulocytes take up Probe (**8**) and up regulate CD11b. Probe (**8**) signal is abrogated by co-incubation with Sivelestat (100 µM), whereas CD11b up regulation is not. MFI = Geometric mean fluorescent index. Statistical analysis by Kruskal-Wallis with post-hoc Dunn, *P < 0.05, **P < 0.01, exact multiplicity adjusted p values are shown with the figure.

Neutrophil activation refers to a series of internal and external neutrophil phenotypic changes that occur as a circulating quiescent neutrophil responds to a noxious stimulus. In their activated state they are recruited to sites of inflammation and contribute to host defence but also to host damage through production and release of serprocidins. We investigated if probe (**8**) could be utilised to profile peripheral blood neutrophils with increased intracellular activity of serprocidins in a rapid flow cytometric assay in whole human blood. The ability of probe (**8**) to label intracellular serprocidin activity in 10 µL of whole blood was analysed using a no-wash no-lyse assay (Fig. 6). This also demonstrated the cell specificity of probe (**8**) as no fluorescence was observed in monocytes or lymphocytes. Data correlated with the assessment of neutrophil activation by up-regulation of CD11b, a cell surface marker known to be associated with neutrophil activation and severity of disease^24^. Mean fluorescent signal from Probe (**8**) increased by a factor of 9.1 from the control to activated condition, while CD11b was up regulated by a factor of 3.7. There was also a relative knockdown when cells and Probe (**8**) were co-incubated with Sivelestat and, as expected, no knockdown of CD11b up-regulation in the presence of Sivelestat.

## Discussion

Branched fluorescent compounds for the detection of serprocidin activity have been reported^15^, and linear FRET seprocidin sensors have been previously reported^25^. The work described here incorporates both strategies onto a single exemplar fluorescent molecule. The incorporation of a multivalent scaffold adds both intracellular targeting and enhanced signal to noise upon de-quenching. We have demonstrated the super-silent nature of this probe in its uncleaved state and the rapid de-quenching that occurs specifically in the presence of a physiologically and pathologically important group of enzymes. We demonstrate the ability of the probe to delineate the spatial arrangement of serprocidin activity both intra and extracellularly. Molecular imaging of intracellular serprocidin activity in live cells by probe (**8**) is distinct from reporters that tag PMNs by membrane lipophilic insertion^26^ and recently reported HNE probes that were used to demonstrate the presence of inactive neutrophil elastase on extracellular extrusions (neutrophil extracellular traps)^27^. Conventional intracellular targeting with poly-arginine linking to linear FRET peptides^28^ for enzyme detection may have issues of cell toxicity and have inherently high background fluorescent levels. Probe (**8**) showed no cellular toxicity during the biological assays and demonstrated no red cell membrane toxicity (Supplemental Fig. S5). We do not indicate any selectivity for one serprocidin over another. We use HNE as the exemplar serprocidin to indicate utility during *in vitro* assays, and the serine proteases inhibitors used in this work are not specific to human neutrophil elastase activity^29,30^ so no specificity towards this particular serine protease can be claimed. *In vivo*, there is no distinct extracellular release of individual serprocidins from primary azurophilic granules during neutrophil activation and there is potential utility in using serprocidins as a whole to rapidly identify the activated neutrophil. It is conceivable that the non-specific AAPV sequence described herein could be exchanged for a more specific peptide sequence if the detection of individual serprocidin activity were desired, and fluorescent probes with unique peptide sequences have been previously used to delineate spatial arrangement of individual serprocidins in isolated neutrophils^31^.

We also assessed the capability of the probe to rapidly profile human PMNs using flow cytometry (FC) using a no-wash no-lyse methodology ideally suited for rapid turnaround in the clinical environment. FC has been used to quantify protease activity by the cleavage of fluorescent compounds incorporated onto the surface of microspheres^32^ and simple peptide fluorophore conjugates have been used to label specific cells for detection^33^. All these approaches require extensive sample processing and fixation and have not been geared towards near patient testing. To our knowledge specific cell associated protease activity has never been detected in rapid, unprocessed clinical samples using FC. Although clinical situations where FC is deployed routinely are relatively infrequent^34^, following the development of smaller and more robust FC technologies^35^, and a move towards consensus design of clinical FC studies^36^, bespoke mechanistic probes might be employed at the bedside of patients to rapidly stratify by means not previously available.

## Methods

All amino acids, Aminomethyl Polystyrene Resin (1.23 mmol/g, 100~200 mesh,1% DVB) and Rink Amide Linker were purchased from GL Biochem (Shangai) Ltd and NovaBiochem. 5(6)-carboxyfluorescein was from NovaBiochem and Oxyma from Apollo Scientific. Commercially available reagents were used without further purification.

Analytical reverse-phase high-performance liquid chromatography (RP–HPLC) was performed on an Agilent 1100 system equipped with a Discovery C18 reverse-phase column (50 × 4.6 mm, 5 μm) with a flow rate of 1 mL/min and eluting with H_2_O/CH_3_CN/HCOOH (95/5/0.05) to H_2_O/CH_3_CN/HCOOH (5/95/0.05), over 13 min, holding at 95% CH_3_CN for 2 min, with detection at 254 and 495 nm and by evaporative light scattering.

Semi-preparative RP–HPLC was performed on an Agilent 1100 system equipped with a Phenomenex Prodigy C18 reverse-phase column (250 × 10 mm, 5 μm) with a flow rate 2.5 mL/min and eluting with 0.1% HCOOH in H_2_O (A) and 0.1% HCOOH in CH_3_CN (B), with a gradient of 5 to 95% B over 25 min and an initial isocratic period of 2 min.

Electrospray ionization mass spectrometry (ESI–MS) analyses were carried out on an Agilent Technologies LC/MSD Series 1100 quadrupole mass spectrometer (QMS) in an ESI mode. High-resolution mass spectra were recorded on a Bruker SolariX Fourier transform ion cyclotron resonance mass spectrometer (FT-MS). MALDI TOF spectra were acquired on a Bruker Ultraflextreme MALDI TOF/TOF with a matrix solution of sinapic acid (10 mg/mL) in H_2_O/CH_3_CN/TFA (50/50/0.1).

### Synthesis of monomer (6)

Monomer (**6**) was prepared in six steps (Scheme S1) with an overall yield of 15%, following the procedure that is described in Avlonitis *et al*.^15^.

### Solid Phase Synthesis

Aminomethyl polystyrene resin (1.23 mmol/g, 1% DVB, 100–200 mesh) was derivatized using 4-[(2,4-dimethoxyphenyl)-(Fmoc-amino)methyl]phenoxyacetic acid (Fmoc-Rink amide linker). The Fmoc-Rink-amide linker (3 mmol, 3eq) was dissolved in DMF (0.1 M) and ethyl(hydroxyimino)cyanoacetate (Oxyma, 3 mmol, 3eq) was added and the mixture was stirred for 10 min. *N*,*N*′-Diisopropylcarbodiimide (DIC, 3 mmol, 3eq) was then added and the resulting mixture was stirred for a further 2 min. The solution was added to aminomethyl polystyrene resin (1 mmol, 1eq) and shaken for 3 hours at room temperature. The resulting resin was washed with DMF (×3), DCM (×3) and MeOH (×3). The coupling reaction was monitored by the Kaiser test^37^.

### Fmoc deprotection

To the resin (1 mmol) pre-swollen in DCM was added 20% piperidine in DMF (10 mL) and the reaction mixture was shaken for 10 min. The solution was drained, and the resin was washed with DMF (×3), DCM (×3) and MeOH (×3). This procedure was repeated twice.

### Isocyanate coupling

To the linker-loaded resin (625 mg, 1.0 mmol), pre-swollen in DCM (10 mL), was added a solution of isocyanate (**6**) (2.7 g, 3.0 mmol), DIPEA (0.5 mL, 3.0 mmol) and DMAP (7 mg, 0.6 mmol) in a mixture of DCM/DMF (1:1, 10 mL) and the mixture was shaken overnight and the reaction monitored by a quantitative ninhydrin test. The solution was drained, and the resin was washed with DMF (3 × 20 mL), DCM (3 × 20 mL) and MeOH (3 × 20 mL) and ether (3 × 20 mL). (3 × 20 mL).

### Dde deprotection

To the resin (200 mg, 0.32 mmol), pre-swollen in DCM (5 mL), was added 2% hydrazine in DMF (3 mL) and the reaction mixture was shaken for 2 h. The solution was then drained, and the resin was washed with DMF (3 × 20 mL), DCM (3 × 20 mL) and MeOH (3 × 20 mL).

### Amino acid coupling

A solution of the appropriate Fmoc-amino acid (3 mmol, 3 eq) (Fmoc-Ahx-OH, Fmoc-Lys(Dde)-OH, Fmoc-Val-OH, Fmoc-Pro-OH, Fmoc-Ala-OH, Fmoc-β-Ala-OH) and ethyl(hydroxyimino)cyanoacetate (Oxyma) (3 mmol, 3eq) in DMF (0.1 M) was stirred for 10 min. N,N′-Diisopropylcarbodiimide (DIC) (3 mmol, 3 eq) was then added and the resulting solution was stirred for a further 2 min. The appropriate solution was then added to the resin (1 mmol), pre-swollen in DCM, and the reaction mixture was shaken for 3 hours at room temperature. The solution was drained and the resin washed DMF (×3), DCM (×3) and MeOH (×3). All coupling reactions were monitored by the Kaiser test (primary amines) and the chloranil test for secondary amines^38^.

### Methyl Red coupling

A solution of 2-(4-dimethylaminophenylazo)benzoic acid (Methyl Red) (3 mmol, 3 eq) and Oxyma (3 mmol, 3 eq) in DMF (0.1 M) was stirred for 10 min. DIC (3 mmol, 3 eq) was then added and the resulting solution was stirred for a further 2 min. The appropriate solution was then added to the resin (1 mmol, 1 eq), pre-swollen in DCM, and the reaction mixture was shaken for 3 hours at room temperature. The solution was drained, and the resin washed DMF (×3), DCM (×3) and MeOH (×3). The coupling reaction was monitored by the Kaiser test, and repeated if not quantitative.

### 5(6)-carboxyfluroscein coupling

A solution of 5(6)-carboxyfluorescein (3 mmol, 3 eq) and Oxyma (3 mmol, 3 eq) in DMF (0.1 M) was stirred for 10 min. DIC (3 mmol, 3 eq) was then added and the resulting solution was stirred for further 2 min. The appropriate solution was then added to the resin (1 mmol, 1 eq), pre-swollen in DCM, and the reaction mixture was shaken for 3 hours at room temperature. The solution was drained, and the resin washed DMF (×3), DCM (×3) and MeOH (×3). The coupling reaction was monitored by the Kaiser test and repeated until complete. After the coupling the resin was washed with 20% piperidine in DMF to remove any fluorescein phenol esters^39^.

### Cleavage from the resin

To the resin pre-swollen in DCM was added the cleavage mixture TFA/TIS/DCM (90/5/5) (1 mL/100 mg resin) and the mixture was shaken for 3 hours at room temperature. The resin was removed by filtration and the resin was washed with the cleavage mixture once (0.5 mL). The combined filtrates were added dropwise to cold diethyl ether to precipitate crude (**8**). This was collected by centrifugation and the diethyl ether was decanted. This solid was washed with diethyl ether and the procedure was repeated three times.

### Purification of probe (8)

Purification of the probe was performed on a Phenomenex Prodigy C18 reverse-phase column (250 × 4.6 mm, 5 μm) with a flow rate 2.5 mL/min and eluting with 0.1% HCOOH in H_2_O (A) and 0.1% HCOOH in CH_3_CN (B), with a gradient of 5 to 95% B over 25 min and an initial isocratic period of 2 min (*t*_*r*_ = 21.2 min).

### Analysis and Characterization of probe (8)

Analysis of the probe was performed on a Discovery C18 reverse-phase column (50 × 4.6 mm, 5 μm) with a flow rate 1 mL/min with detection at 254 nm and by evaporative light scattering (*t*_*r*_ = 7.86 min, purity >99% based on 245 nm). FTMS *m/z* Calc mass for C_215_H_264_N_38_O_46_ 1372.9922 [M + 3/3]^+^; found 1373.04119 [M + 3/3]^+^

### Use of human tissue

Whole blood from healthy volunteers was collected according to Lothian Research Ethics Committee approval (#08/S1103/38). Healthy volunteers provided written informed consent prior to collection of blood. All work with human tissue samples was carried out according to the relevant guidelines and regulations. The Principal Investigator at the Centre of Inflammation Research, University of Edinburgh approved all experimental protocols.

### Cell isolation and culture

Human peripheral blood leukocytes were prepared as previously described^40^. Briefly, citrated blood was centrifuged at room temperature for 20 min at 350 g, and platelet-rich plasma was removed. Leukocytes were separated from erythrocytes by dextran sedimentation using 0.6% dextran T500 (Pharmacia, Milton Keynes, UK), and the leukocyte-rich layer was then fractionated using isotonic Percoll (Pharmacia). Neutrophils were harvested from the 68%/81% interface.

*Pseudomonas aeruginosa* (J3284-clinical isolate from a patient with ventilated associated pneumonia) was grown on agar plates and stored at 4 °C. For assays a single colony of bacteria was taken using an inoculating loop and added to 10 ml liquid broth in a 50 ml Falcon Tube. This was transferred to an incubator at 37 °C for 16 hours. Cultures were used as overnight cultures (stationary phase); the culture was centrifuged at 4000 rpm for 5 minutes and pellet resuspended in phosphate buffered saline (PBS pH 7.4, Life technologies, Carlsbad, CA, USA). Following three washes the optical density at 595 nm was measured.

### Fluorescent microplate reader experiments

Probe (**8**) (5 µM) was incubated with HNE in reaction buffer (50 mm Hepes buffer, pH 7.4, 0.75 M NaCl, 0.05% Igepal CA-630 (v/v), all Sigma-Aldrich, Poole, UK) with or without Sivelestat (100 µM, Tocris, Bristol, UK). The time course of fluorescence dequenching was followed for 45 min with a fluorescence microplate reader (excitation 480/20, emission 528/25). The limit of detection was calculated by the mean of probe alone at each time point plus 3x the standard deviation and generating a standard curve by linear regression analysis of the known enzyme concentrations. All experiments were n = 3 and data was analysed by one-way ANOVA with post-hoc analysis by Bonferroni. Neutrophil lysate was prepared by re-suspending 10 × 10^6^ PMN/ml in reaction buffer followed by multiple freeze-thaw cycles using a dry-ice/acetone bath.

### Live Cell Imaging and intra-cellular staining for confocal microscopy

Two experimental protocols were employed. Firstly, isolated neutrophils were stained with a nuclear dye to demonstrate an overview of cellular probe signal. Secondly, neutrophils were stained with a cytoplasmic dye and combined with fluorescently labelled bacteria to demonstrate the vesicular localisation of the cellular probe signal. All live imaging was performed in IMDM (Life Technologies). Approximately 150,000 neutrophils were seeded onto glass coverslips pre-coated with 10 μg/ml fibronectin (Sigma-Aldrich). Where Sivelestat (100 µM) was included, cells were pre-treated prior to activation and continued presence of inhibitor was ensured throughout assays where appropriate.

To record overall neutrophil cellular signal, Syto-82 nuclear stain (Life technologies, final concentration 2.5 µM) was added to adherent neutrophils that were activated by the addition of either Calcium Ionophore (A21387 10 µM, Sigma-Aldrich) or Pseudomonas aeruginosa. Following activation NE probe was added to a final concentration of 5 µM. To record the vesicular localisation of probe (**8**), Pseudomonas aeruginosa, at one optical density, were labelled with PKH (CellVue claret far red fluorescent cell linker kits, Sigma-Aldrich) according to the manufacturers instructions and added to adherent neutrophils. Incubation proceeded for 30 minutes at 37 °C prior to the addition of Calcein-AM red-orange cytoplasmic dye (500 nM, Life technologies). Following a single wash with IMDM probe (**8**) was added to a final concentration of 5 µM. Imaging proceeded within 10 minutes.

A laser-scanning confocal imaging system (LSM510; Carl Zeiss, Jena, Germany), incorporating an upright Axioskop FS2 microscope (63× objective) was used for image acquisition and processing. Exposure to 488 nm light was limited to 5% of the maximum laser power in order to minimize toxicity. In all cases, images were obtained without Kalman averaging and typically with a pixel dwell time of 3.2 µs with a pinhole diameter corresponding to 1 Airy unit. Pinhole diameters were adjusted to give optical Z-sections of equivalent depths, corresponding to 1 Airy unit for the longest excitation wavelength and images were acquired sequentially. Fluorescein was excited with a dedicated 488 nm line and emitted light detected with a meta detector (500–530 nm). Syto nuclear and calcein cytoplasmic dyes were excited with a dedicated 543 nm line and emitted light detected with a meta detector (560–600 nm). PKH labelled bacteria were excited with a dedicated 633 nm line with emitted light detected with a meta detector (650–705 nm). Fields of view were chosen blindly based on Syto 82 or Calcein-AM labelling as appropriate.

### Whole blood cytometric analysis

Whole blood was analysed using a modification of the no-wash no-lyse protocol published by Li *et al*.^41^. Whole blood was collected from healthy volunteers and gently mixed with citrate (final concentration 0.38%). Within 10 minutes 10 µL of citrated whole blood were added to pre-mixed solutions containing 0.2 µg each of CD45 PE/Cy7 (clone H130, Biolegend, London, UK) and CD11b APC (clone ICRF44, Biolegend) made up to 100 µL with IMDM in 2 mL microtubes. Antibody quantity was determined by prior titration experiments (data not shown). For activated conditions pre-mixed solutions also contained final concentrations of Cytochalasin B (5 µg/ml, Sigma-Aldrich) and *N*-formyl methionyl-leucyl-phenylalanine (fMLP, 500 nM, Sigma-Aldrich). Sivelestat 100 µM was added where appropriate. Samples were incubated at 22 °C and agitated at 300 rpm for 30 minutes in the dark using an orbital plate shaker. Probe (**8**) was then added to a final concentration of 5 µM and returned to the plate shaker for 10 minutes under the same conditions. To stop the reaction 1.4 ml of IMDM was added to each microtube and samples were stored on ice pending cytometric analysis, which took place within 2 hours. Samples were analysed with a FACSCalibur (Becton Dickenson, San Jose, CA, USA) flow cytometer. The cytometer was aligned weekly using Calibrite 3, APC and 8 peak beads (Becton Dickenson) to calibrate scatter and fluorescence parameters. Voltages remained consistent throughout the study period. The cytometer was thresholded on forward scatter to eliminate debris. Samples were analysed at a flow rate to yield approximately 5–15 CD45 positive events per second for a total duration of 4 minutes (CD45 positive events typically in the region of 1000). Fluorescence parameters were collected using a four-decade logarithmic scale. PE/Cy7-CD45 positive events were selected initially to separate leukocytes from red blood cells. Lymphocytes, monocytes and granulocytes were independently distinguished by their light scattering properties and by their CD45 expression. Only cells pooled from the appropriate gates from both CD45/SSC and FSC/SSC plots were subsequently analysed for FL1 (probe (**8**)) and FL4 (CD11b APC) fluorescence. Data analyses were performed using Flowjo v10.0.7 (Treestar, Ashland, OR, USA). For each fluorescent parameter the geometric mean was recorded for each condition in each donor. Samples were repeated in triplicate for each individual donor and pooled to yield a point estimate for each condition and each donor. The point estimates from four different donors were pooled to yield an overall mean point estimate for each condition ± SEM. Differences among conditions were compared using Kruskal-Wallis with post-hoc Dunn correction.

### Membrane toxicity

Probe (**8**) membrane toxicity was assessed using erythrocyte haemolysis. Isolated erythrocytes remaining after leukocyte separation were diluted 1:5 with PBS without cations (Life technologies) and 50 µL of this red cell suspension were added to wells of a 96-well plate. Working concentration of probe **(8)** (5 µM), or working concentration x2, was added to the relevant well. In separate wells dimethyl sulphoxide (DMSO, Sigma-Aldrich) at either 0.5% or 1% was added to reflect final DMSO vehicle concentrations in these two concentrations of probe (**8**), data not shown. Positive control wells contained Triton X-100 (0.4%, Sigma-Aldrich) and negative control wells contained PBS w/o only. Conditions were repeated in triplicate for each blood donor. The 96-well plate was incubated for one hour at 37 °C in a humidified environment. Following incubation, 180 µL of PBS without cations was added to each well and the plate was centrifuged at 2500 rpm for 10 minutes. 100 µL of supernatant from each well was removed and aliquoted into clean wells. Absorbance at 350 nm was recorded using a plate reader (Synergy H1 hybrid reader, BioTek, Potton, UK). Triplicate repeats were pooled to yield an overall mean point estimate ± SEM of haemolysis that was expressed as a percentage of the positive control.

### Statistical analyses

All statistical analyses were performed in Prism v6 (GraphPad Software, La Jolla California USA).

## Electronic supplementary material

Supplementary information
