# Supplementary material for: Super-silent FRET Sensor Enables Live Cell Imaging and Flow Cytometric Stratification of Intracellular Serine Protease Activity in Neutrophils
Source: Sci Rep. 2018 Sep 10;8:13490. doi: 10.1038/s41598-018-31391-9 (PMC6131393; doi:10.1038/s41598-018-31391-9)
Supplement: Supplementary file 1 — Supplementary information [file 41598_2018_31391_MOESM1_ESM.docx]

Super-silent FRET Sensor Enables Live Cell Imaging and Flow Cytometric Stratification of Intracellular Serine Protease Activity in Neutrophils

Thomas H Craven, Nicos Avlonitis, Neil McDonald, Tashfeen Walton, Emma Scholefield, Ahsan R Akram, Tim S Walsh, Chris Haslett, Mark Bradley* & Kevin Dhaliwal*

Supplemental Materials

**Figure S1**: Synthesis of the tri-branched monomer (**6**)

## Figure S2. Identification of the HNE cleavage site of probe (8). Probe (8) 2μM was incubated with HNE (1mg/mL) in HEPES buffer at 37 ºC for 1 hour. The cleavage site (Val-Lys) was identified by MALDI TOF MS analysis (Figure S3)

**Figure S3.** Probe (**8**) 2μM was incubated at 37 ºC for 1h with HNE (1mg/mL) in HEPES buffer (**A**) Full MALDI-TOF spectra m/z 2135.9 [M+H]^+^; (**B**) MALDI-TOF spectra (experimental and theoretical zoom) m/z 2135.9 [M+H]^+^, 2157.7 [M+Na]^+^; (**C**) Structure of the cleavage product.

******

**Figure S4:** Activation of freshly isolated human PMN with *Pseudomonas aeruginosa,* labelled with Syto-82, followed by addition of probe (**8**) and imaged at t+10 minutes. Syto 82 also labels the PMN nucleus. Punctate fluorescent signal from de-quenched probe can be seen throughout the cytoplasm of the PMN. Scale bar 10 µM.

******

**Figure S5.** Toxicity of probe (**8**) assessed by red cell haemolysis. Lysis determined by free haemoglobin 350 nm light absorption. Triton X (0.4%) causes complete membrane lysis. Probe (**8**) at 5 µM and 10 µM showed no additional membrane toxicity over vehicle.
